# Land suitability assessment for supporting transport planning based on carrying capacity and construction demand

**DOI:** 10.1371/journal.pone.0246374

**Published:** 2021-02-08

**Authors:** Long Li, Gaoru Zhu, Dafang Wu, Honglei Xu, Peifang Ma, Jie Liu, Zhaocheng Li, Yinjie He, Chenghui Li, Pan Wu

**Affiliations:** 1 School of Geography and Remote Sensing, Guangzhou University, Guangzhou, China; 2 Planning and Research Institute of the Ministry of Transport, Beijing, China; 3 School of Public Administration and Policy, Renmin University of China, Beijing, China; Gebze Teknik Universitesi, TURKEY

## Abstract

With the rapid global urbanization, the unlimited increasing transportation infrastructure has met the needs of urban expansion, but it has caused a series of ecological problems lacking consideration of ecological conservation. The land suitability assessment for supporting transport planning based on carrying capacity and demand for construction is an effective way to promote urban socioeconomic development and ecological conservation. Therefore, we constructed a logical framework of resources and environment supporting, traffic construction demand driving, and ecological protection red line and basic farmland constraining, and applied the analytic hierarchy process (AHP), GIS, three-dimensional magic cube method, and gravity model to evaluate the suitability of expressway development in Sichuan Province, China. The results showed that the spatial difference in the carrying capacity of resources and environment and the demand for expressway construction was relatively high in Sichuan, and those in eastern cities were even higher. The land suitability for supporting transport planning was relatively high, and the suitable areas with a grade from 8 to 10, accounted for 20.77% of the total study area, which could almost meet the demand for transportation infrastructure construction. The land suitability performed a circle structure with Chengdu as the core and gradually decreasing to the periphery. Overall, this study adds new insights to transport planning reform in other similar regions around the world and can provide important references for regional development planning and environmental protection.

## 1 Introduction

Land space is the region on which human beings depend for survival and development, and also the carrier of socioeconomic activities [1]. Spatial planning is a public management tool for spatial allocation and distribution of resource elements. It aims to balance environmental protection and the needs of economic development to achieve sustainable economic and social development. It is authoritative, comprehensive, strategic, regional, and restrictive [2]. The current planning system in China is complex and imperfect, and there have been some problems such as conflicts of various types of planning content, poor linkage of various departments, and complex work processes [3, 4], resulting in prominent human-land conflicts, unbalanced development of urban and rural areas, low efficiency of land space development, and insufficient ecological protection, among others [5–7]. The newly proposed national spatial planning integrates the functions of various planning departments and improves the coordination mechanism among various departments. By implementing the "one map" of the planning, it can unify and manage efficiently natural resource and makes overall arrangements for spaces for ecological protection, production, and live to achieve the governance system modernization in China [8]. As special planning under the national spatial planning, transport planning has an important influence on the spatial form, structure, organization, scale, and function of the ecological space, urban, and agricultural space [9]. How to scientifically evaluate the land suitability for supporting transport planning and more effectively connect with the national spatial planning is necessary to achieve the balance between transportation, urban development and environmental protection.

Transportation is a basic and leading industry, which is closely related to the allocation of land resources, spatial shape shaping, spatial structure, spatial organization, and regional main function guidance [10]. Transport planning has changed the relationship between time and space, which is an important driving force that changes the pattern of land space. 20% of land space in the world have been directly affected by the transportation network [11]. Traditional transport planning faces the following problems: First of all, the traditional thinking that it mainly builds more longer facility increment to meet the needs of urban economic construction and expansion [12], has resulted in waste of traffic land, resources extensive use, and environmental pressure increase, among others [13, 14]. Secondly, the previous transport planning has aimed to meet the needs of economic development, paying more attention to the feasibility of engineering construction, while the concept of ecological protection has been still lacking. And the comprehensive protection of land space is less considered [15]. The aim of the national spatial planning based on ecological civilization is to restrict the disorderly expansion of urban, and to ensure that the ecological and agricultural space is not largely occupied [16]. Thus, transport planning not only meets the needs of urban development but also reflects the idea of ecological management and control. Besides, the traditional traffic network evaluation mainly includes environmental impact assessment, effect evaluation of the established road network [17], or evaluation of routes selection for the feasibility stage [18], which analyzes the conflict between transportation infrastructure and ecologically sensitive targets, towns, basic farmland, and other natural and social geographic elements to make roads optimization. However, it has been rare to consider the constraints of ecological environment and resources on transportation infrastructure from the source, failing to thoroughly imply the concept of environmental protection [19]. Furthermore, the technical content of urban planning at various levels in the past has been scattered and incomplete, poorly connecting with land use planning, so that it could not meet the requirements of traffic land and other lands’ interactive development [20].

The evaluation of resources and environment carrying capacity and the land suitability assessment (referred to as the double evaluation) are important foundations for improving the scientific nature of national spatial planning. The carrying capacity of resource and environment refers to the maximum scale and capacity of the regional resource and environment system to bear various social and economic activities under a certain period and space within a specific socioeconomic level and production and lifestyle, and under the premise that the resource structure and environmental function meet the needs of sustainable development [21]. Studies related to carrying capacity begin with ecology and early studies focus on individual carrying capacity, such as land, water resources, and agricultural carrying capacity [22]. Due to the increasingly prominent ecological and environmental problems, the carrying capacity evaluation gradually extends to the comprehensive evaluation of resources, environment, and ecological environment [23]. Widely used methods mainly include principal component analysis, system dynamic method, ecological footprint, emergy analysis, multi-criteria decision making, the theory of shortboard, and analytic hierarchy process (AHP) [24–26]. Research is widely used in many fields including pasture and wildlife management, aquaculture, post-disaster reconstruction, industrial planning, and structure, among others [22, 27]. The application of the double evaluation in national spatial planning has become a new trend in Chinese carrying capacity research [28]. However, relevant research has rarely applied it to the field of transport planning.

The land suitability refers to the suitability of land space for different functions and uses such as agricultural production, urban construction, and ecological protection under specific development goals and needs within a certain period and space. Research methods mainly include the AHP, multi-element space superposition, and the three-dimensional magic cube method [29–31]. Among them, the three-dimensional magic cube method has been widely used, which is a method of matrix combination with multi-dimensional input and single-dimensional output. It can achieve a good balance between highlighting significant factors and taking into account the overall score, which is conducive to achieving regional multi-target sustainable development [32]. The perspective of the study has changed from a single element to a multi-target evaluation such as meeting land development or agricultural production standards [33]. To improve the accuracy and rationality of national spatial planning, the land suitability evaluation based on the carrying capacity of resources and the environment has become a new trend in this field [34]. The index system for evaluating urban construction is relatively rich, but there are few studies on the suitability assessment for supporting transport planning, meanwhile, insufficient consideration has been given to the environmental constraints and requirements of transportation infrastructure.

Sichuan Province, located in southwestern China, is in a very important position in the Chinese "One Belt, One Road" strategy and the strategy of western development. It is an ecological barrier on the upper reaches of the Yangtze River and an important growth pole for the development of the Yangtze River Economic Belt in China. However, its location disadvantages and complex terrain have caused poor transportation conditions, resulting in unbalanced regional development, the concentration of poor population, and aggravated ecological and environmental problems. The conflict between the urgency of economic development and ecological safety protection has become more prominent. Therefore, with Sichuan as the study area, we evaluated the land suitability through the double evaluation to balance the relationship between ecological environment protection and regional economic development. Base on existing studies, this study established a logical framework of resources environment supporting, traffic construction demand driving, and ecological protection red line and basic farmland constraining, which mainly included the following four goals: (1) to evaluate the resource and environment carrying capacity of transportation construction from four aspects, including land resources, ecological environment, climate and natural disasters, (2) to comprehensively evaluate the demand for traffic construction based on current traffic conditions and the strength of connections between urban nodes, (3) to divide areas with a different restricted degree under the management and control requirements of the ecological protection red line and basic farmland, (4) to comprehensively evaluate the land suitability for supporting transport planning based on the above three points.

## 2 Materials and methods

### 2.1 Study area

**Fig 1:**
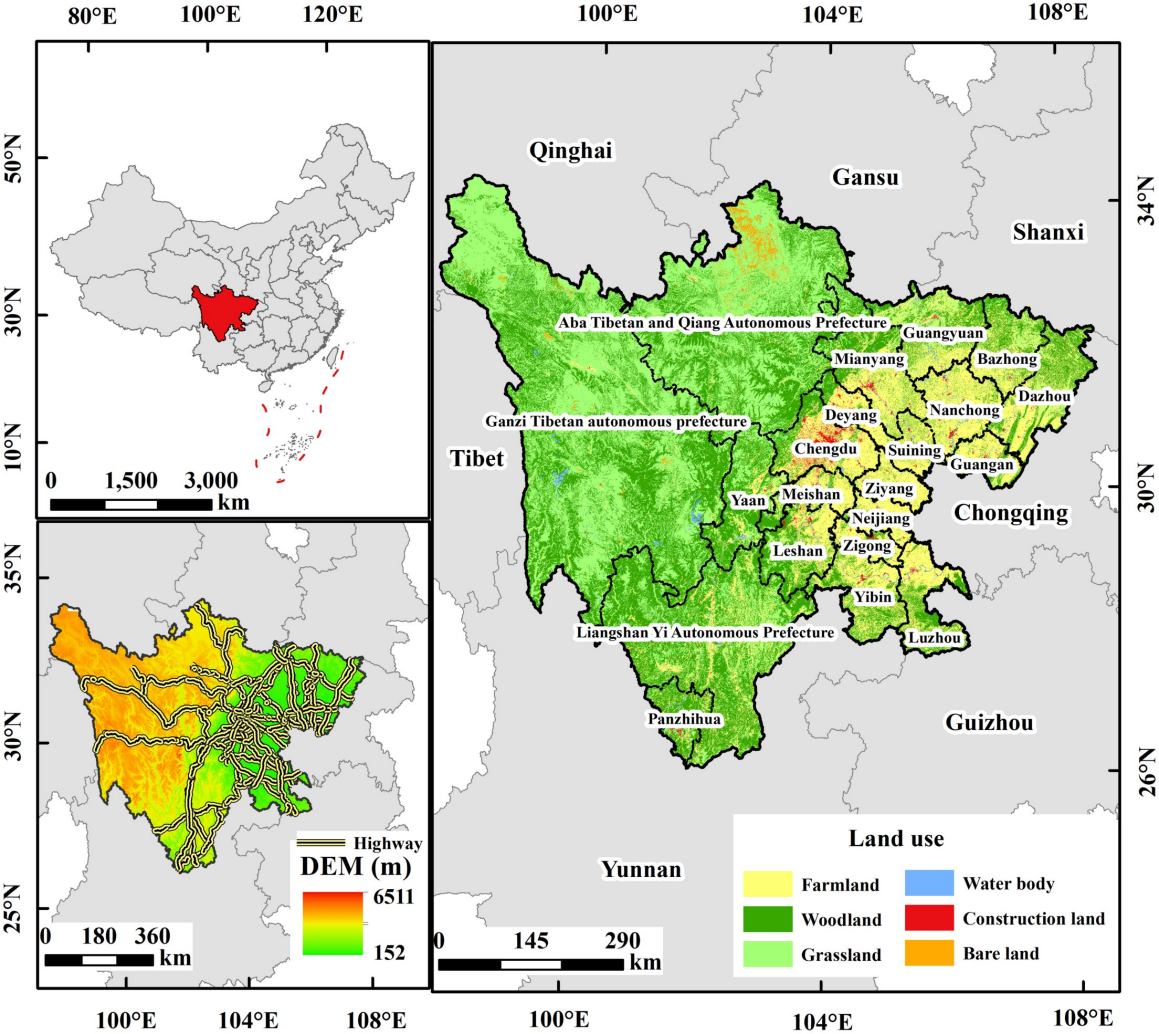
Land cover and location of the study area. The image was obtained by using ArcGIS 10.2 based on the DEM data from the Geo-spatial Data Cloud (http://www.gscloud.cn), land cover data from Resource and Environmental Science Data Center of the Chinese Academy of Sciences (http://www.resdc.cn/), and highway data from National Basic Geographic Information System Database (http://www.ngcc.cn/ngcc/). The base map outline was obtained by using ArcGIS 10.2 based on the Service Center of Standard Map (http://bzdt.ch.mnr.gov.cn/) and the number of the permission is GS (2020) 4621.

Sichuan Province is located in Southwest China (26°03’34°19’N, 97°21’108°12’E) (Fig 1), with a total area of 486,000 km^2^. Located in the transition zone between the first-level Qinghai-Tibet Plateau and the third-level plain of the middle and lower reaches of the Yangtze River, the terrain is characterized by high altitudes in the west and low altitudes in the east, forming a complex landform that combines mountains, hills, plains, basins, and plateaus. It has a mid-subtropical humid climate with an average annual precipitation of 900-1200mm, and temperatures between 16–18°C. The area is rich in natural resources such as minerals, animals, and plants, as well as tourist resources such as scenic spots. Dominated by the Yangtze River system, it is an important area for water conservation and ecological barrier on the Yangtze River.

By the end of 2019, the GDP reached 4661.58 billion yuan, and the resident human was 83.75 million. The urbanization rate reached 53.79%, indicating a relatively backward economic development condition and a low level of urbanization. Meanwhile, the development level between urban and rural areas and the traffic condition between eastern and western areas differed greatly. By the end of 2015, the total mileage of the comprehensive transportation network reached 331,000 km. The traffic conditions are relatively backward in the western minority areas, old revolutionary base areas, and concentrated contiguous areas with extreme poverty. Due to underdeveloped traffic conditions and low economical levels, Sichuan Province urgently needs to build and improve a comprehensive transportation network system.

### 2.2 Data sources

Land cover data whose resolution is 30m was obtained from Resource and Environmental Science Data Center of the Chinese Academy of Sciences (http://www.resdc.cn/). The elevation data was derived from the Geo-spatial Data Cloud (http://www.gscloud.cn) SRTM90m data. Water data was derived from China’s 1:4 million water system distribution data set. Data such as temperature and precipitation were from the China Meteorological Data Network (http://data.cma.cn). The geohazard data come from the geohazard bulletin issued by the Ministry of Land and Resources. The data of the fault zone come from the 1:500,000 geological map of Sichuan Province from the Geological Bureau. The data of the central node come from the "National Comprehensive Three-dimensional Transportation Network Planning Research Report" (2020) of the Ministry of Transport. Socio-economic data such as population, GDP, administrative level, among others come from the "Statistical Yearbook of Sichuan Province" (2019). The transportation infrastructure data such as roads and railways come from the National Basic Geographic Information System database (http://www.ngcc.cn/ngcc/).

### 2.3 The research framework

**Fig 2:**
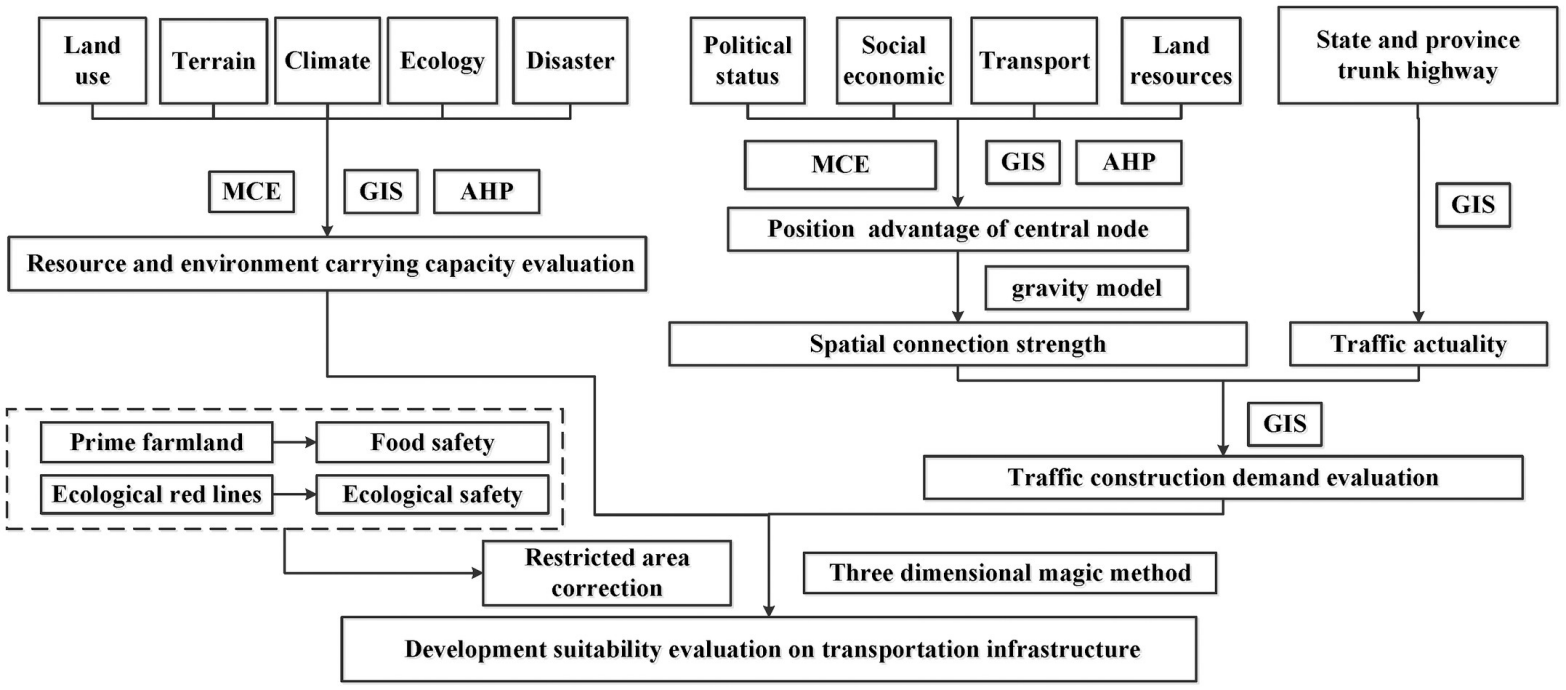
The research framework.

To evaluate the land suitability, a four-step procedure was developed in this study as shown in Fig 2. Firstly, the carrying capacity of the resource environment was assessed to determine the carrying capacity on traffic construction. Then we analyzed the strength of the land space connection and current traffic conditions to determine the demand for construction. Thirdly, different levels of restricted development areas were delineated from the perspective of food security and ecological security. Finally, the land suitability for supporting transport planning was estimated based on three aspects above.

### 2.4 Resources and environment carrying capacity assessment

#### 2.4.1 Establishment and weight of index system

Firstly, based on the local environmental conditions of Sichuan Province and the influencing factors that support the construction of transportation infrastructure, nine indicators were selected from five dimensions, including land use, topography, ecological resources, climate conditions, and natural disasters, to establish the index system of resources and environment carrying capacity on transportation. Among them, land resources were carriers that carry all human economic and social activities and played a fundamental role in transportation construction. Terrain conditions played a decisive role in the convenience of transportation construction. The smoother the terrain, the easier it is to carry out related engineering constructions and save costs. Ecological resources had a strong restriction on transportation construction. The richer the ecological resources, the higher the importance of ecological protection, and the weaker the capacity to carry transportation infrastructure. Temperature and precipitation played an important role in road conservation and natural restoration. Natural disasters were often easy to cause damage to the road structure and cause a reduction in service life, which is not conducive to the sustainable use of roads. Secondly, the AHP method was used to assign index weights, mainly including four steps. Firstly, the hierarchical structure model was established. The second step was to establish the judgment matrix to compare the importance of each index. Thirdly, the consistency test was carried out on the judgment matrix. Finally, the weight of the index was calculated based on the importance. The specific index system and weights were shown in Table 1.

**Table 1:** Resources and environment carrying capacity evaluation index system.

Guidelines level	Index	Number	Weight	attributes
Land use	Land cover	a	0.19	Neutral
Terrain	Elevation	b	0.11	Negative
	Slope	c	0.15	Negative
Ecological resources	Water conservation	d	0.13	Negative
	Quantity of inhabited species	e	0.09	Negative
Climatic conditions	Temperature	f	0.07	Positive
	Precipitation	g	0.05	Positive
Natural disaster	Types of geological hazards	h	0.13	Neutral
	Fracture zone buffer zone	i	0.08	Negative

#### 2.4.2 Level classification

By judging whether the index value was continuous, indexes were divided into two types, namely continuity indicators and type indicators. The continuity indicator refers to the index with definite value, which was in the continuous state. The type indicator refers to the index with approximate range and without definite value, which was in the discrete state. For continuity indicators, the natural breakpoint method was used to divide each indicator into 5 levels. For type indicators, each indicator was classified through relevant technical standards or existing index grading methods [35, 36]. The threshold range of every indicator was shown in Table 2. The higher the score, the higher the carrying capacity of each single element on the transportation infrastructure.

**Table 2:** Resources and environment carrying capacity index grading assignment.

Number	Unit	Threshold				
		1	2	3	4	5
a	-	Water area	Forest land	Cultivated land	Grassland	Construction land, unused land
b	m	≥4042	[3132,4042)	[2062,3132)	[1014,2062)	[186,1014)
c	°	≥35	[25,35)	[15,25)	[8,15)	[0,8)
d	-	Extremely important	Highly important	Moderately important	Lowly important	Not important
e	-	5,6	3,4	2	1	0
f	°C	[-14.64,1.39)	[1.39,5.47)	[5.47,10.56)	[10.56,15.66)	≥15.66
g	mm	[289,549)	[549,727)	[-727,889)	[889,1044)	≥1044
h	-	Debris flow	Landslide	Collapse	Ground subsidence, unstable slope	Non-hazardous area
i	km	[0,10)	[10,20)	[20,30)	[30,40)	≥40

Note: a: land cover; b: elevation; c: slope; d: water conservation; e: quantity of inhabited species; f: temperature; g: precipitation; h: types of geological hazards; i: fracture zone buffer zone.

#### 2.4.3 Evaluation method

Firstly, the multi-criteria analysis was used to measure the comprehensive carrying capacity of resources and environment on the transportation infrastructure [37]. The score of resource and environment carrying capacity was obtained by the weight in Table 1 and the score in Table 2. Secondly, the quantile method was used to divide the carrying grade into 10 levels. The higher the level, the higher the carrying capacity of the resource environment on the transportation infrastructure.

$$Y=\sum_{i=1}^{n}X_{i}\times w_{i},$$ (1)

where *Y* is the score of resource and environment carrying capacity, *i* is the number of the single indicator, *n* is the quantity of indicators, *w*_*i*_ is the weight of *i*-th indicator in Table 1, *X*_*i*_ is the score of *i*-th indicator in Table 2.

### 2.5 Evaluation of the demand of traffic land construction

#### 2.5.1 Evaluation of the interregional strength of connection

(1) Evaluation on the location advantage of central nodes. Firstly, 12 indicators were selected from the four aspects including political strategy, social economy, transportation, and land space to establish the index system of location advantage of central nodes. Among them, the political strategy included two indexes, namely administrative level, and strategic importance, which reflected the political attraction between the central nodes. The social economy included two indexes, namely population, and GDP, which reflected the level of regional socio-economic. The target of transportation included three indicators, namely, highway, civil aviation, and railway, reflecting the convenient conditions of regional transportation. The land space includes two indexes, namely natural resources and spatial equilibrium, which reflected the local conditions of land and resources. Secondly, the weight of each factor was calculated by the AHP. Finally, based on the multi-criteria analysis, the comprehensive score of each central node was calculated by multiplying the score of each index by the weight, so as to comprehensively evaluate the location advantage of each central node. Specific indicators and weights were shown in Table 3.

**Table 3:** Index system and weight of location advantage of central nodes.

Guidelines level	Standard level	Index level	Weight
Political strategy	Administrative status	Administrative level	0.5
	Strategic status	Strategic importance	0.5
Society and economy	Population	Household population	0.5
	Economic	GDP	0.5
Transportation	Highway	National highway density	0.25
		Ordinary national highway density	0.2
	Civil aviation	Airport class	0.15
		Distance from the nearest airport	0.25
	Railway	Railway density	0.15
Land space	Natural resources	Land area	0.4
	Spatial equilibrium	Distance from bordering counties	0.35
		Relief	0.25

(2) Measurement of interregional connection strength. The gravity model is a classical model for analyzing the strength of spatial connection and interaction. It can reflect the law of the formation and evolution of spatial structure from multiple dimensions and has been widely used in urban research and other fields [38]. There are many ways to improve the gravity model. In this study, the strength of each central node interaction was calculated by the modified gravity model. The traditional gravity model has used population and GDP scale to assess the quality of a city [39]. However, with the improvement of transportation level, scientific and technological development, and constant adjustment of policies, the scale of population and GDP cannot fully reflect the comprehensive strength of a city [40]. The intensity of spatial connection between central nodes was mainly reflected in four aspects including political- strategic position, socio-economic level, transportation conditions, land, and resource endowment. Therefore, the interaction intensity between central nodes was measured based on the score of the location advantage of the central node mentioned above, and the distance between nodes. The gravity model was calculated as shown in eq 2.

$$G_{ij}=KM_{i}M_{j}/f(D_{ij}),$$ (2)

where *G*_*ij*_ is the connection strength between node *i* and node *j*, *M*_*i*_ and *M*_*j*_ represent the score of location advantage of node *i* and node *j*, respectively, *f* (*D*_*ij*_) is the straight-line distance parameter, *D*_*ij*_ is the straight-line distance between node *i* and node *j*, *K* is the empirical coefficient and k is 1 [41]. *G*_*ij*_ reflects the degree of correlation of the traffic location between nodes. The larger the value, the closer the connection between the pair of nodes, and the greater the demand for the construction of traffic and transportation network, and vice versa.

After obtaining the connection strength between the nodes, the first 20% effective pairs of the connection strength were selected and set a buffer zone with a width of 10km to get the total score of the spatial connection strength in Sichuan. Based on the quantile method, the score was divided into 10 levels from high to low. The higher the level of the area, the closer the link with other areas, the stronger the interaction, and the more urgent the transportation infrastructure construction.

#### 2.5.2 Evaluation of current conditions of roads

Based on the technical standards of highway engineering issued by the Ministry of Communications, the current layout of national and provincial trunk highways in Sichuan was divided into five technical levels from high to low, namely, expressways, first-level roads, second-level roads, third-level roads, and fourth-level roads. 10 km-wide buffer zones of different technical levels of roads were built as shown in eq 3.

$$B_{{}_{x}}^{i}=\{x:d(x,R_{i})\leq 10\},$$ (3)

Where *B* is scores of buffers, *x* is the unit of evaluation, *i* is the technical level of roads, *d* is the distance between the unit of evaluation and roads, *R*_*i*_ is *i* th-level roads. Scores of buffers of different technical levels of roads were shown in Table 4.

**Table 4:** Scores of buffers of different technical levels of roads.

Technical levels of roads	Scores of buffers
Expressways	5
First-level roads	4
Second-level roads	3
Third-level roads	2
Fourth-level roads	1

The total score was obtained by overlaying scores of different buffers. Based on the quantile method, the current condition of roads was divided into 10 levels from high to low. The higher the level, the more convenient the traffic conditions, and the higher the demand for road upgrading.

#### 2.5.3 Comprehensive evaluation of the demand for traffic construction

Firstly, the demand for traffic construction was calculated as shown in eq 4.

$$D_{x}={\text{SICS}}_{x}+{\text{SCCR}}_{x},$$ (4)

Where *D*_*x*_ is the score of the demand for traffic construction, *x* is the unit of evaluation, SICS_*x*_ is the score of interregional connection strength, SCCR_*x*_ is the score of current conditions of roads. Secondly, the demand for traffic construction in Sichuan was divided into 10 levels from high to low through the quantile method. The higher the level of the area, the greater the demand for the construction of highways and other transportation networks.

### 2.6 Delimitation of restricted areas for land space development

According to the managing and controlling requirements of various nature reserves, ecological protection red line, and basic farmland, areas were divided into high-restricted areas, medium-restricted areas, low-restricted areas, and unrestricted areas. Standards for different grades of restricted development zone delimitation were shown in Table 5.

**Table 5:** Standards for dividing areas with different degrees of restriction.

Restriction level	Area
High restriction	Core area and buffer zone of nature reserve; core protected area of national park; first-level area of drinking water source protected area
Medium restriction	Scenic areas including super protected area, first-level protected area, and core area; forest park including ecological protected area and core area; super protected area, geological heritage protected area; wetland park protected area; world cultural and natural heritage core protected area; core protected areas of nature reserves
Low restriction	National parks, nature reserves, scenic spots, forest parks, geological parks, wetland parks, drinking water source protected areas, world cultural and natural heritage sites, ecological protection red line except for high and middle restricted areas, and basic farmland
No restriction	Other areas

### 2.7 Land suitability assessment for supporting transport planning

#### 2.7.1 Establishment of a three-dimensional magic cube model

**Fig 3:**
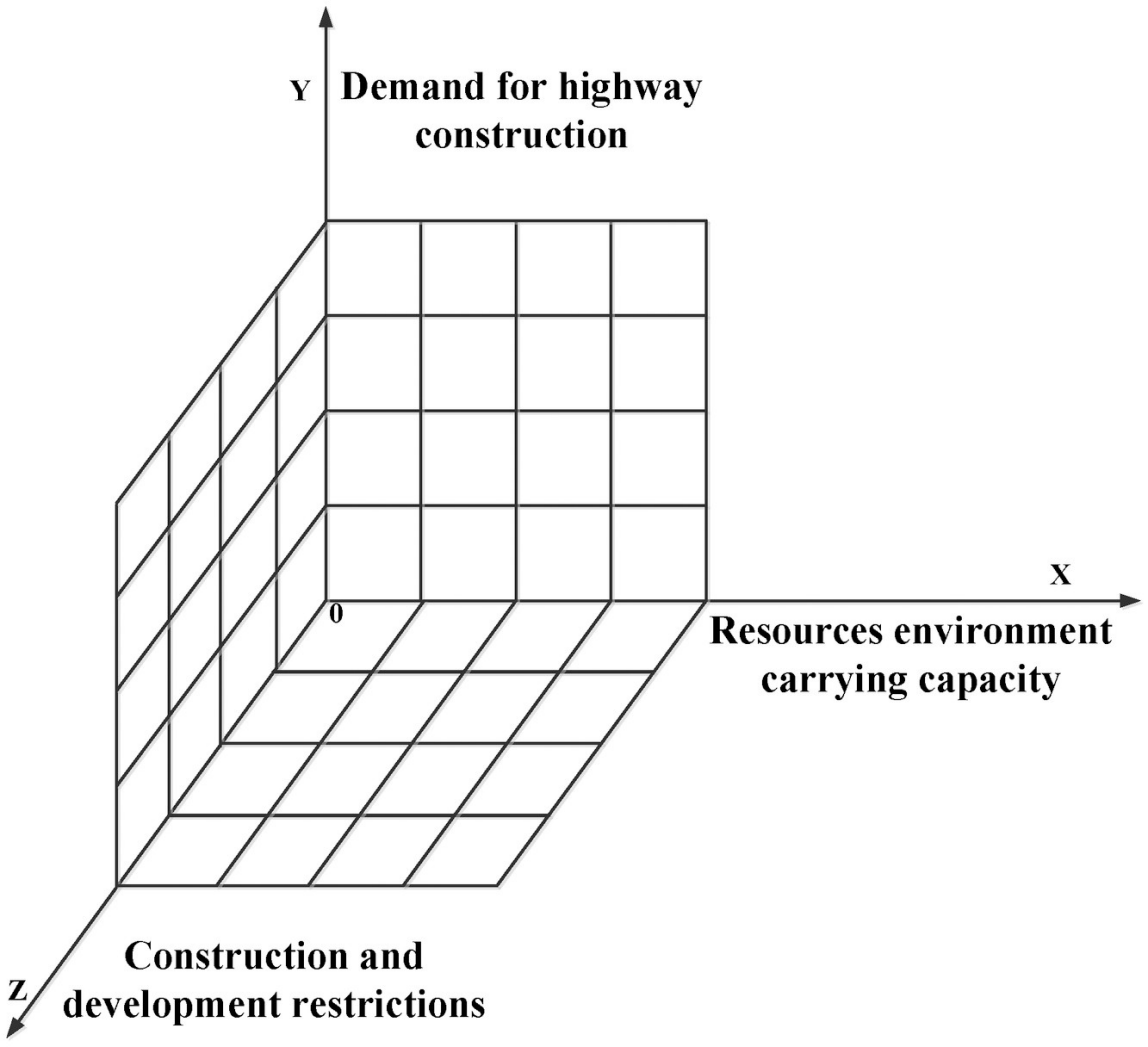
3D Cube of carrying capacity, construction demand, and restricted development.

The land suitability was composed of three major elements including resources and environmental carrying capacity, the demand for traffic construction, and restricted area of land space development, which corresponded to the X-axis, Y-axis, and Z-axis in the three-dimensional magic cube. The carrying capacity of resources and environment represented the supporting capacity of natural resources on transportation infrastructure. The demand for traffic construction reflected the strength of the spatial connection between cities and the necessity of construction. Based on the consideration of food security and ecological security, the restricted area of land space development constrained the intensity and layout of highway construction to vary degrees. The schematic diagram of the three-dimensional magic cube model was shown in Fig 3.

#### 2.7.2 Preliminary land suitability evaluation

By coupling results of resources and environment carrying capacity and the demand for traffic construction, the preliminary suitability was obtained. Based on the coupling criteria of the two, the result was divided into 10 levels. The specific coupling standards for the two were shown in Table 6.

**Table 6:** Coupling standard of the carrying capacity and construction demand.

		**Traffic construction demand**									
		1	2	3	4	5	6	7	8	9	10
**Resource and environment carrying capacity**	1	1	1	1	2	2	2	3	3	3	4
	2	1	2	2	2	3	3	3	4	4	5
	3	2	2	3	3	3	4	4	5	5	5
	4	3	3	3	4	4	5	5	5	6	6
	5	3	4	4	5	5	5	6	6	7	7
	6	4	5	5	5	6	6	7	7	7	8
	7	5	5	6	6	7	7	7	8	8	8
	8	6	6	7	7	7	8	8	8	9	9
	9	7	7	7	8	8	8	9	9	10	10
	10	7	8	8	8	9	9	10	10	10	10

Note: The larger the value, the darker the color, indicating that the higher the land suitability.

#### 2.7.3 Revised land suitability assessment

According to the constraining degree of land space development, the aforementioned preliminary land suitability was revised, and the weight for high-restricted areas, medium-restricted areas, low-restricted areas, unrestricted areas was assigned 40%, 60%, 80%, 100%, respectively. The preliminary result was multiplied by the weight and rounded to obtain the revised suitability. The higher the level, the more suitable for the construction of the transportation infrastructure.

### 2.8 Analysis of the suitability of expressway development

Firstly, a 600m buffer zone was established on both sides of the expressway (including projects that have been built and will be built) in 2020. Secondly, the zonal tool was used to count scores of the suitability per unit area of the buffer zone. Finally, ranking the statistical scores from high to low, the suitability of expressway development was divided into 5 levels based on the natural breakpoint method, which were high suitability, relatively high suitability, moderately suitable, relatively low suitability, and low suitability.

## 3 Results

### 3.1 Carrying capacity of resources and environment

**Fig 4:**
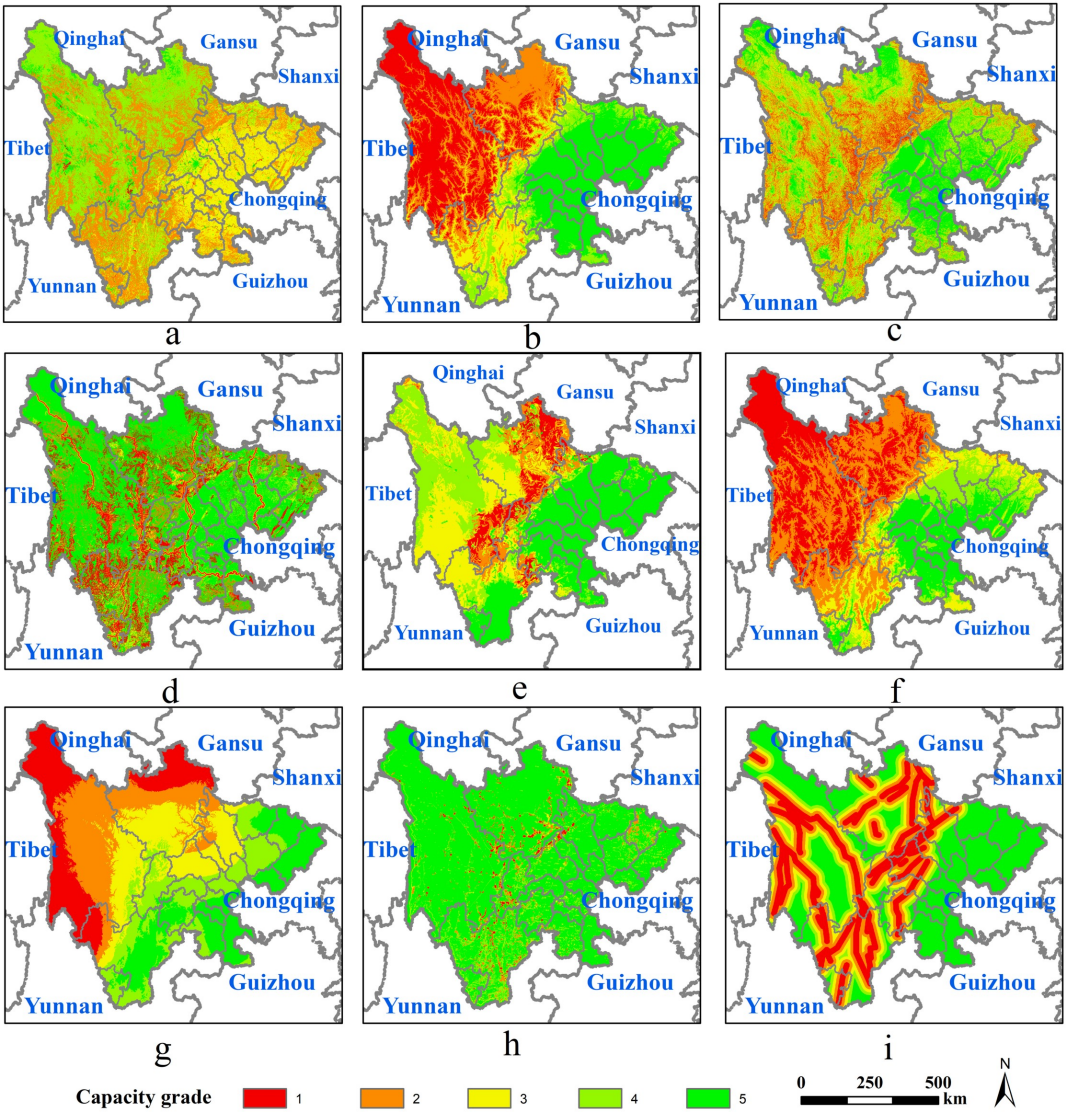
Grades of single-element carrying capacity of resources and environment. The image was obtained by using ArcGIS 10.2 through the open-access data process. The base map outline was obtained by using ArcGIS 10.2 based on the Service Center of Standard Map (http://bzdt.ch.mnr.gov.cn/) and the number of the permission is GS (2020) 4621.

Firstly, as shown in Fig 4, the distributed characteristics of carrying grades of elevation, slope, temperature, and precipitation tended to coincidence, which was high in the east, low in the west, and gradually decreased from east to west. Secondly, the distributed characteristics of carrying grades of land use, water conservation, species, and fracture zone tended to similarity, and high-grade areas were mainly distributed in the eastern and northwestern regions. The low-grade areas presented a distribution of long and narrow strip in the middle areas. At last, high-grade geological hazards were concentrated in the eastern and western regions, and the rest were scattered in the central portion.

**Fig 5:**
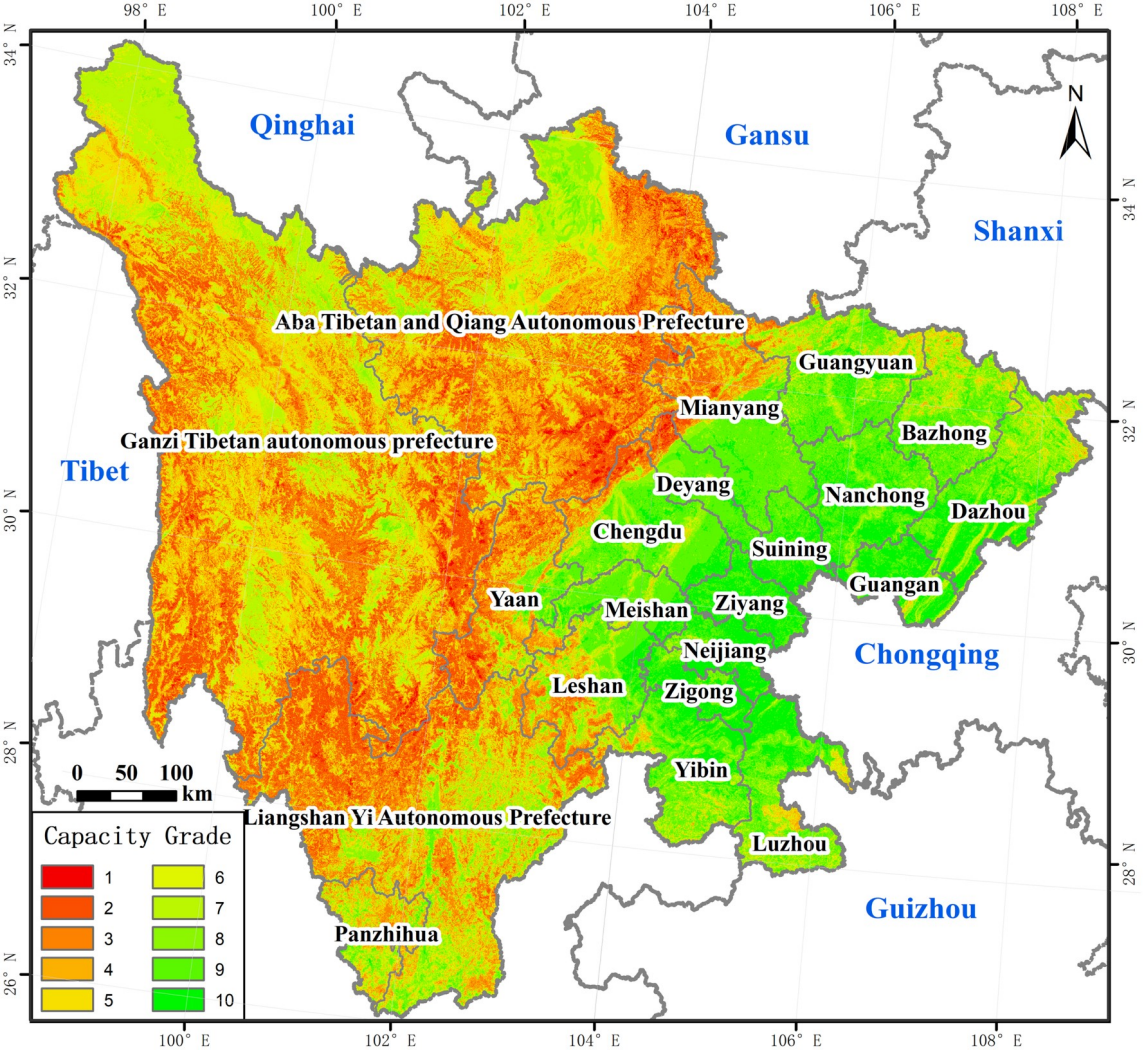
Comprehensive carrying capacity of resources and environment. The image was obtained by using ArcGIS 10.2 through the data process. The base map outline was obtained by using ArcGIS 10.2 based on the Service Center of Standard Map (http://bzdt.ch.mnr.gov.cn/) and the number of permission is GS (2020) 4621.

As shown in Fig 5, the overall grade of carrying capacity of resources and environment in Sichuan was relatively high. Its spatial distributed characteristics were high in the east, north, and south, and low in the middle and west sides. As shown in Table 7, areas with a grade from 8 to10 had a higher carrying capacity and were mainly distributed in the eastern and north-south marginal areas. They covered 141520.87km^2^, accounting for 29.04% of the total study area. These areas had good land resources and climate conditions, which were beneficial to the natural restoration of highways. Meanwhile, it had the advantages of a low probability of natural disasters occurrence and gentle terrain, which was beneficial to traffic construction. Areas with a grade from 5 to 7 were ordinary, which was mainly located around the low-grade area or scattered in the eastern region. They covered 160999.93km^2^, accounting for 33.04% of the total study area. The terrain, climatic conditions, and local conditions in these areas were better than those of areas with a grade from 1 to 4, and they were also potential areas for future traffic construction. The carrying grade in these three ethnic autonomous prefectures in the west was relatively low, most of which were in the range of 1 to 4. They covered 184777.10km^2^, accounting for 37.92% of the total study area, where natural disasters occurred frequently and there was a more serious impact on traffic safety. Meanwhile, these areas had characteristics of many species, high altitude, steep terrain, and difficult traffic construction.

**Table 7:** Scale and proportion of different grades of carrying capacity.

Carrying grade	Area (km^2^)	Proportion (%)
1	2662.22	0.55
2	45649.22	9.37
3	70401.26	14.45
4	66064.40	13.56
5	62477.54	12.82
6	46767.23	9.60
7	51755.16	10.62
8	38163.04	7.83
9	63471.80	13.03
10	39886.03	8.19
Total	487297.90	100.00

### 3.2 Evaluation of the demand for transportation construction

#### 3.2.1 Location advantage of central nodes

**Fig 6:**
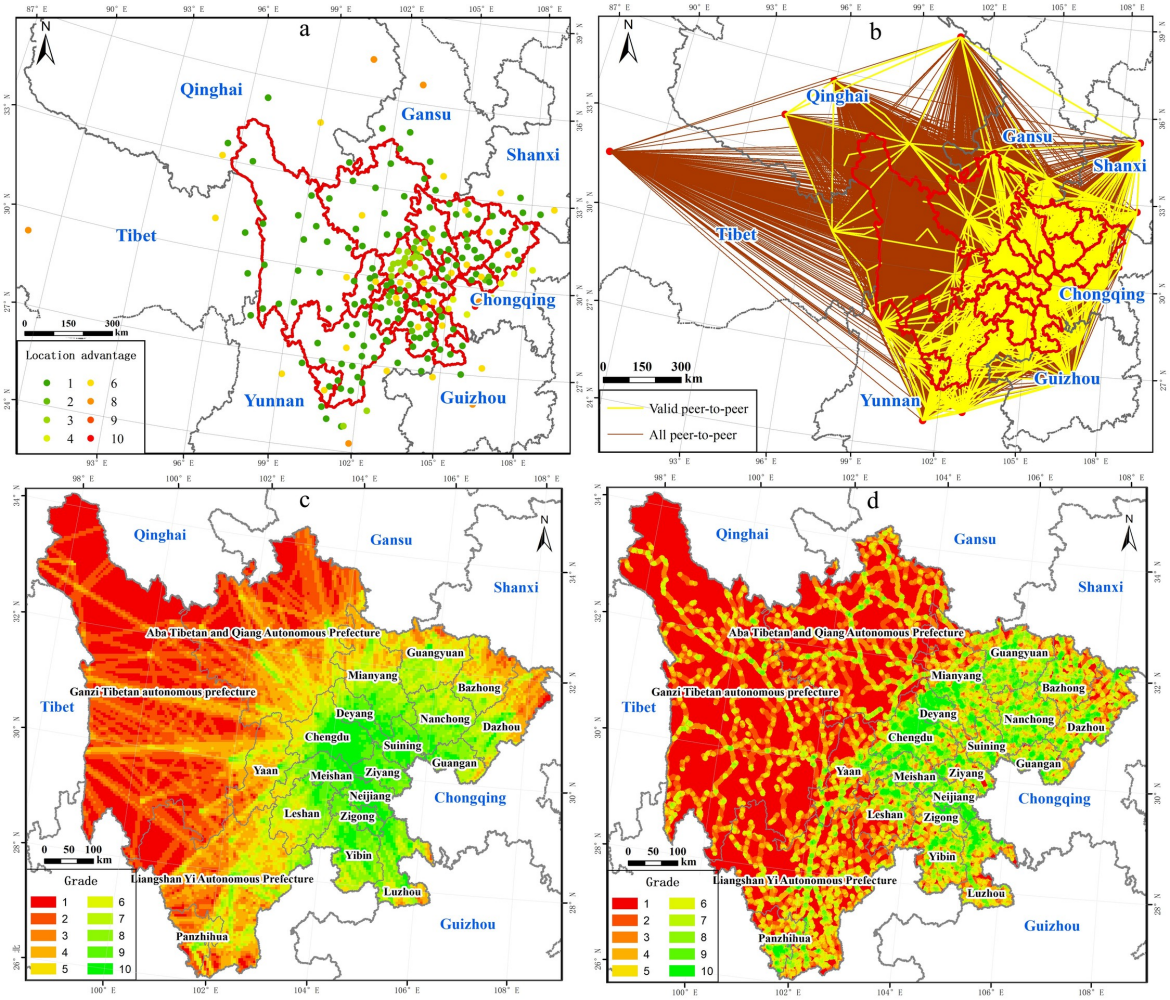
Single-element analysis of the demand for traffic construction. The image was obtained by using ArcGIS 10.2 through the open-access data process. The base map outline was obtained by using ArcGIS 10.2 based on the Service Center of Standard Map (http://bzdt.ch.mnr.gov.cn/) and the number of the permission is GS (2020) 4621. Note: the single-element step is listed as: (a) Location advantage of central nodes; (b) Effective pairs of points; (c) Strength of land space connection; (d) Current traffic conditions.

The location advantage of central nodes was of great significance for the demand for transportation construction. The more important the location of the central node, the more necessary to improve the traffic conditions in the surrounding area to support urban development. As shown in Fig 6a, central nodes with a relatively high superiority degree were gathered around centers of eastern cities and bordering provincial capital centers. The lower-level central nodes were concentrated in the western ethnic autonomous prefectures and districts whose distribution is relatively scattered. Among all central nodes, the central node in Chengdu City whose level was 10 had the highest degree of superiority. These nodes surrounding Chengdu, such as Jianyang City, Mianyang City, and those in the southern region such as Yibin City and Zigong City had relatively high levels, all of which were above level 8. Among central nodes of provincial capital cities in bordering provinces, the location advantage of southern central nodes was higher than in others. Central nodes had the highest degree of superiority in Chongqing City, whose level was 9, followed by Kunming City and Guiyang City, whose level was 8, and the remaining provincial capital city nodes were level 7.

#### 3.2.2 Effective pairs of points selection

Based on the gravity model, a total of 48610 potential pairs of points were calculated, and the range of gravitational value was from 0.9977 to 4922.6082. Sorted by gravitational value, a total of 8176 pairs of points with a gravitational value higher than 60 were selected as effective pairs of points, whose range was from 60.0182 to 4922.6082. As shown in Fig 6b, effective pairs of points were concentrated on the eastern areas and scattered in the western sides. Among the bordering provincial capitals, the quantity of effective point pairs of central nodes was relatively high in Chongqing, Yunnan, and Guizhou, while relatively low in Shanxi, Gansu, Qinghai, and Tibet.

#### 3.2.3 Strength of land space connection

As shown in Fig 6c, the spatially distributed pattern of the strength of land space connection in Sichuan was characterized by high in the east and low in the west. Taking many cities in the east, such as Chengdu City, Deyang City, and Suining City, among others, as the core area, the connection strength decreased gradually to the surroundings. Among them, areas with a level from 8 to 10 were closely connected with surrounding cities, covering 90869.41km^2^, accounting for 18.65% of the total study area. And they were mainly distributed in the eastern region, especially in the cities around Chongqing.

#### 3.2.4 Current traffic conditions

As shown in Fig 6d, current traffic conditions in Sichuan performed ordinarily, with significant differences between the east and west, and conditions in the east were more convenient than those in the west. Among them, there was good transportation infrastructure in these areas with a grade from 8 to 10, which covered 70128.83km^2^, accounting for 14.39% of the total study area, mainly distributed along the Chengdu-Deyang-Mianyang-Guangyuan and Suining-Ziyang-Neijiang-Zigong-Luzhou.

#### 3.2.5 The demand for traffic construction

**Fig 7:**
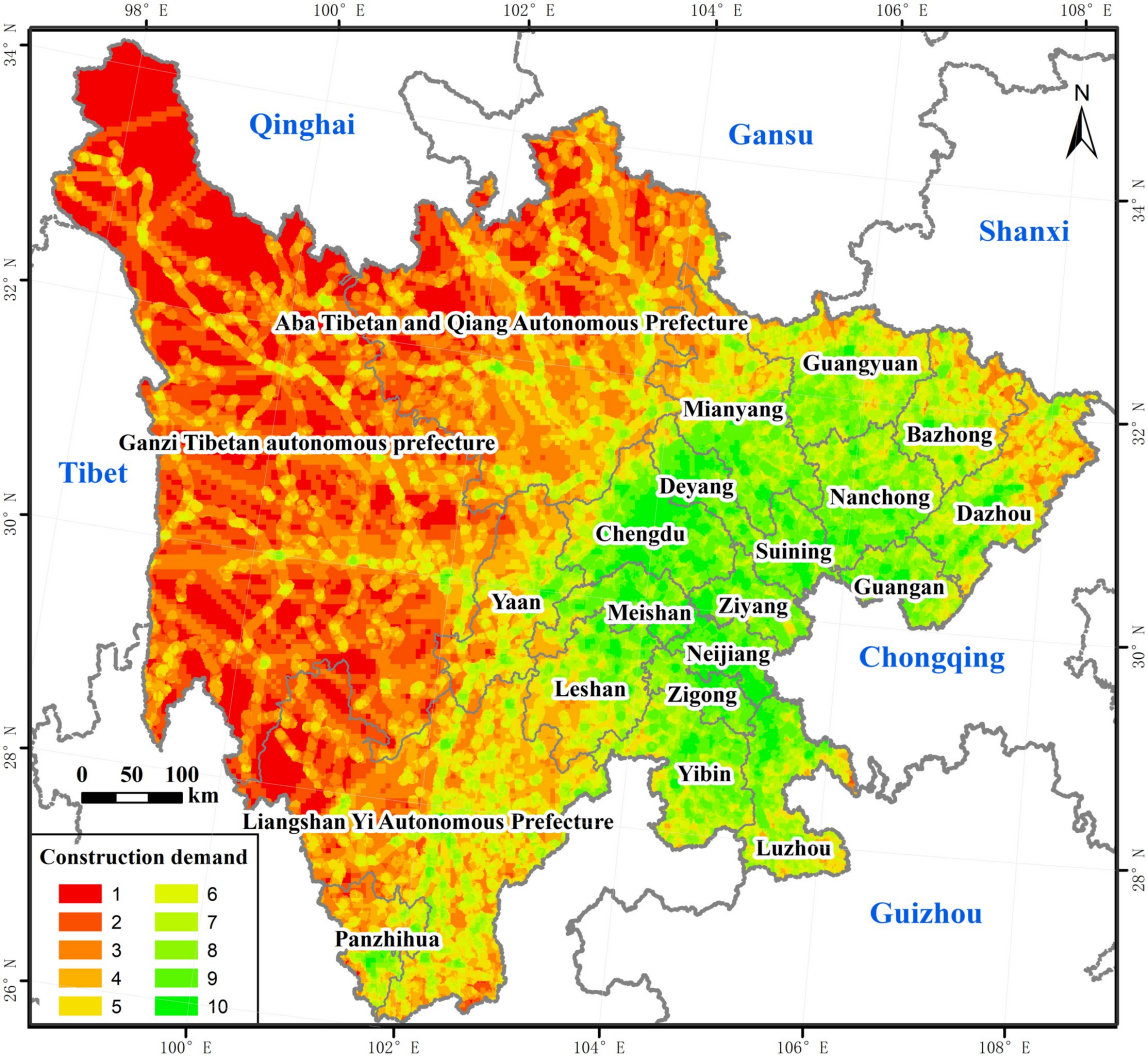
Grades of the demand for traffic construction. The image was obtained by using ArcGIS 10.2 through the open-access data process. The base map outline was obtained by using ArcGIS 10.2 based on the Service Center of Standard Map (http://bzdt.ch.mnr.gov.cn/) and the number of the permission is GS (2020) 4621.

Combining the results of spatial connection strength and current traffic conditions, the demand for traffic construction was calculated. The higher the demand for traffic construction, the higher the necessity of construction. As shown in Fig 7, the overall demand for traffic construction in Sichuan was relatively high, showing a distributed characteristic of high in the east and low in the west. As shown in Table 8, areas with a grade from 8 to 10 had a high demand for traffic construction, which covered 93343.33km^2^, accounting for 19.18% of the total study area. They were distributed in clusters in eastern cities, suitable for the construction of high-grade roads, such as expressway. Areas with a grade from 5 to 7 had a medium demand for traffic construction, which covered 128809.78km^2^, accounting for 26.43% of the total study area. They were mainly distributed in high-demand surrounding areas in a strip shape, which was suitable for the construction of medium-grade roads. Areas with a grade from 1 to 4 had a low demand for traffic construction, which covered 265,024.79 km^2^, accounting for 54.39% of the total study area. They were concentrated in the three ethnic autonomous prefectures in the west, where the demand for high-grade roads was not high, but the construction of low-grade roads or other infrastructures was very urgent.

**Table 8:** Scale and proportion of different grades of traffic construction demand.

Grade	Area (km^2^)	Proportion (%)
1	55513.69	11.39%
2	60558.12	12.43%
3	83379.17	17.11%
4	65573.82	13.46%
5	49556.41	10.17%
6	40550.54	8.32%
7	38702.83	7.94%
8	34896.49	7.16%
9	39583.23	8.12%
10	18983.61	3.90%
Total	487297.9	100.00%

### 3.3 The pattern of restricted development

**Fig 8:**
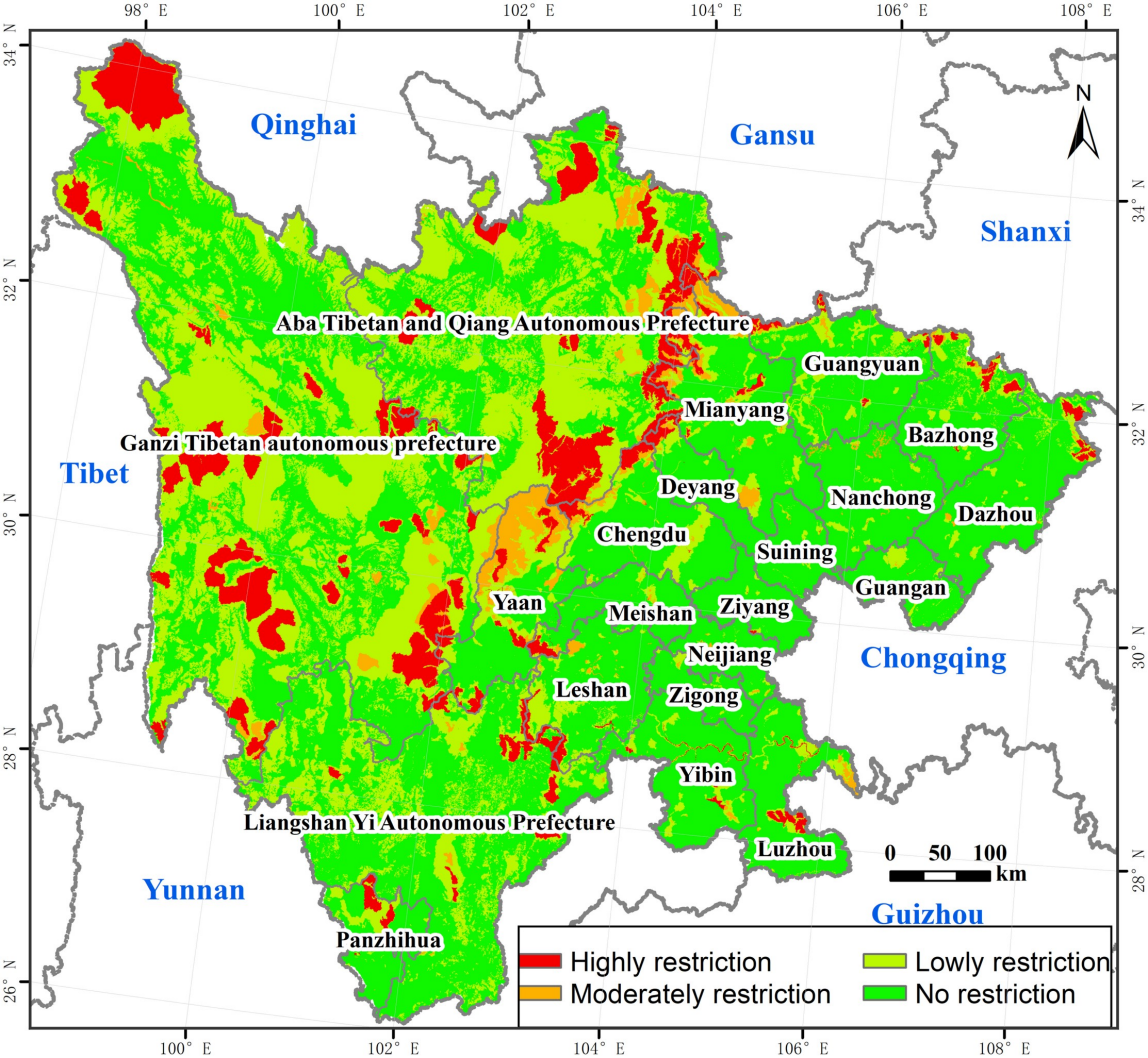
The pattern of restricted development. The image was obtained by using ArcGIS 10.2 through the open-access data process. The base map outline was obtained by using ArcGIS 10.2 based on the Service Center of Standard Map (http://bzdt.ch.mnr.gov.cn/) and the number of the permission is GS (2020) 4621.

As shown in Fig 8, generally, restricted development areas were few in Sichuan, but there were more restrictions on land space in western and central regions. Highly restricted areas covered a 39474.56 km^2^, accounting for 8.10% of the total study area, distributed in a strip between the three ethnic autonomous prefectures and the eastern cities. Moderately restricted areas covered 13861.04 km^2^, accounting for 2.84% of the total study area, mainly distributed in the southern and northeastern parts of Aba Prefecture. Low restricted areas covered 148450.17 km^2^, accounting for 30.46% of the total study area, distributed in the cluster around the highly and moderately restricted areas, concentrated in the autonomous prefectures, and other scattered in the eastern cities. The area of unrestricted areas was 285512.13 km^2^, accounting for 50.60% of the total study area, with obvious distributed characteristics of agglomeration, mainly distributed in the east, northwest, and southwest.

### 3.4 The spatial pattern of the land suitability

**Fig 9:**
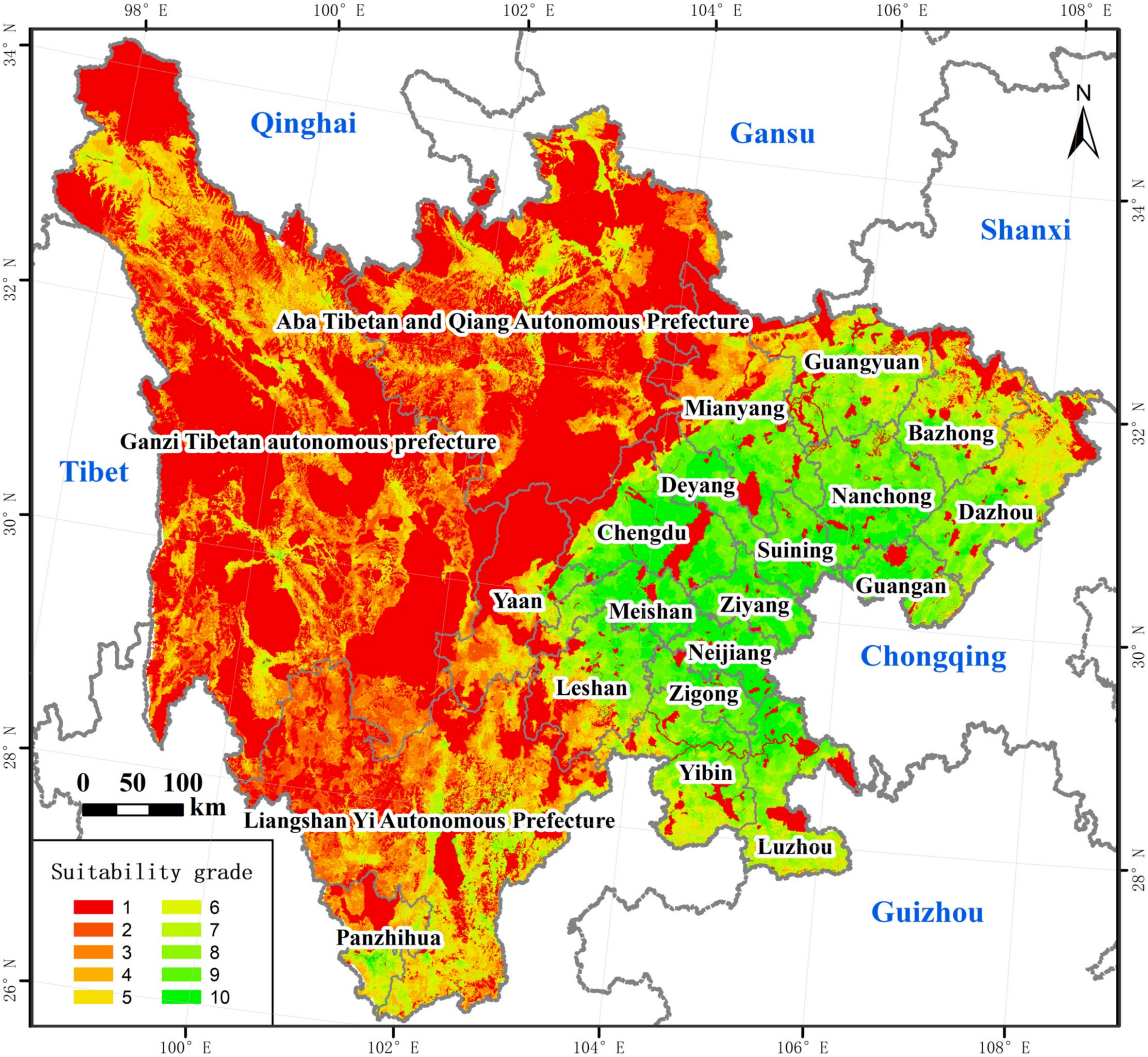
The spatial pattern of the land suitability. The image was obtained by using ArcGIS 10.2 through the open-access data process. The base map outline was obtained by using ArcGIS 10.2 based on the Service Center of Standard Map (http://bzdt.ch.mnr.gov.cn/) and the number of the permission is GS (2020) 4621.

The land suitability for supporting transport planning is of great significance to the site selection of highways and other transportation arterial lines, the scheduling of transportation project construction, and the sustainable use of traffic land. Based on the demand for traffic construction and carrying capacity, combined with restricted development areas, the land suitability in Sichuan was comprehensively evaluated. Sorted by the score from low to high, the results were divided into grades with 1 to 10 through the geometric interval method. As shown in Fig 9, the overall suitability of traffic construction presented a distributed pattern of high in the east and low in the west. The suitability of the eastern areas was relatively high, while relatively low in the western region except for Panzhihua City. As shown in Table 9, the suitability of construction was high in areas with a grade from 8 to 10, where the carrying capacity and the necessity for expressway construction was high. They covered 101203.51 km^2^, accounting for 20.77% of the total study area, concentrated in the eastern cities in a pie shape. Areas with a grade from 5 to 7 were at the moderate grade, covering 87718.12 km^2^, accounting for 18.00% of the total study area, which was scattered mainly in the northwest, southwest, and the periphery of areas with a grade from 8 to 10. Their carrying capacity and construction demand were relatively ordinary. Areas with a grade from 1 to 4 were at the low grade, covering 298376.27 km^2^, accounting for 61.23% of the total study area, concentrated in the central and western areas in clusters. The environmental conditions were poor, and the demand for highway construction was low.

**Table 9:** Scale and proportion of different grades of the land suitability.

Grade	Area (km^2^)	Proportion (%)
1	203560.51	41.77
2	18949.77	3.89
3	31956.66	6.56
4	43909.33	9.01
5	39796.01	8.17
6	25587.24	5.25
7	22334.87	4.58
8	28310.73	5.81
9	42938.38	8.81
10	29954.40	6.15
Total	487297.90	100.00

### 3.5 Analysis of the suitability of expressway development

**Fig 10:**
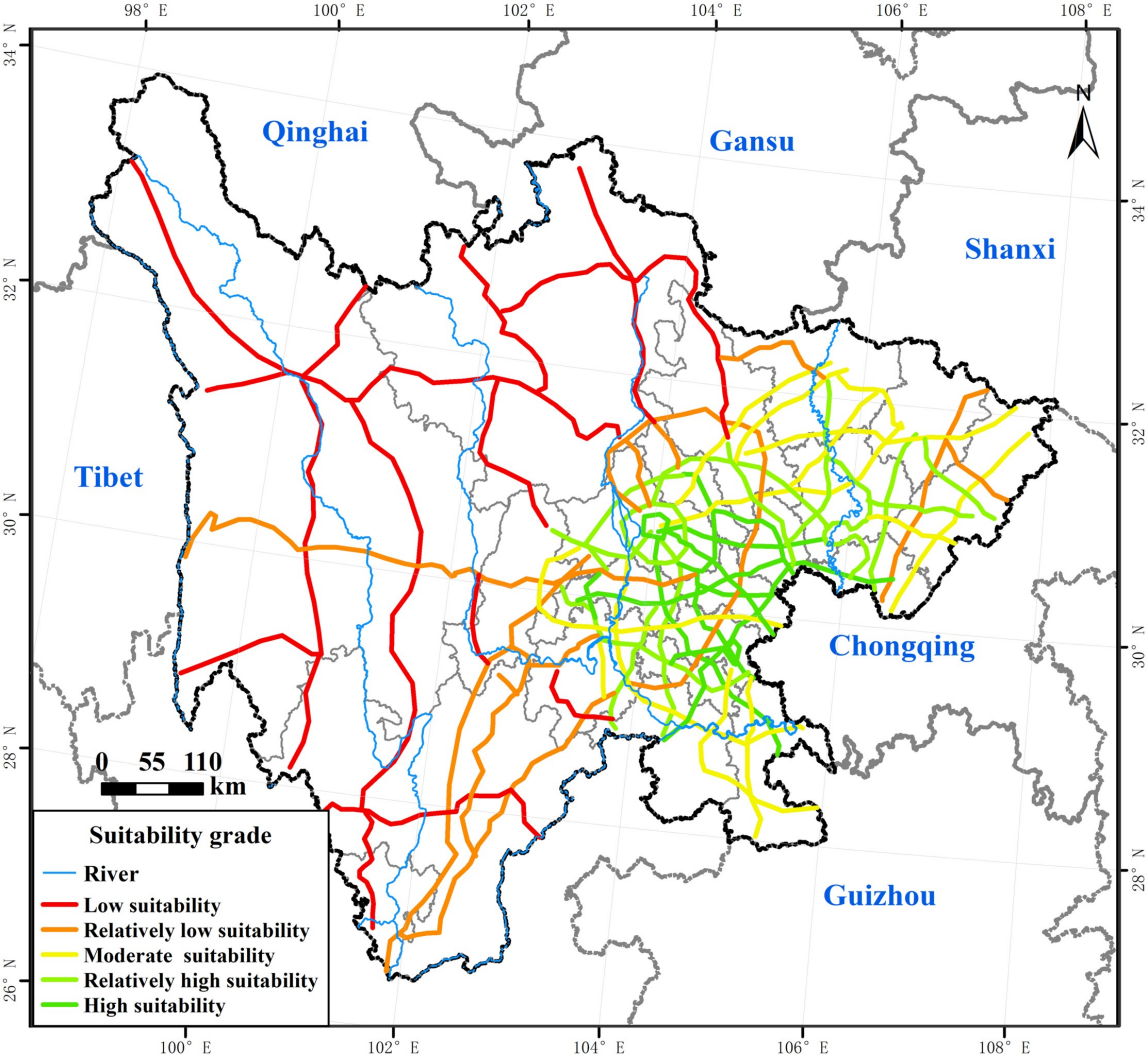
The suitability of expressway development. The image was obtained by using ArcGIS 10.2 through the open-access data process. The base map outline was obtained by using ArcGIS 10.2 based on the Service Center of Standard Map (http://bzdt.ch.mnr.gov.cn/) and the number of the permission is GS (2020) 4621.

Scores of the buffer zone of expressways per unit area ranged from 1.51 to 9.61, showing that the overall development suitability was relatively high in Sichuan. As shown in Fig 10, agglomeration characteristics of the suitability of the expressway in different areas were obvious. With Chengdu as the core and gradually decreasing to the periphery, the suitability of expressway development performed a circle structure with decreasing levels. Among them, the high-suitability highway with the highest score, covered 2227.60 km, accounting for 15.59% of the total length of the highway, which was mainly distributed in the Chengdu Plain and its southeastern area. The area had the advantages of flat terrain, large location advantages, and high economic development. The highway with relatively high suitability for development covered 2518.79 km, accounting for 17.62% of the total length of the highway, mainly distributed in the northeast and southwest of the Chengdu Plain. Although the location and socio-economic conditions were slightly worse than those of the high-suitability highway, the overall conditions were relatively good. The highway that was moderately suitable for development covered 2775.53 km, accounting for 19.42% of the total length of the expressway, mainly distributed outside the relatively suitable area. The highway with relatively low suitability covered 3237.23 km, accounting for 22.65% of the total length of the expressway, mainly distributed in the southwest area. The highway with low suitability covered 3533.46 km, accounting for 24.72% of the total length of the expressway. They were mainly distributed in the three autonomous prefectures of western Sichuan, where the terrain was flat and the strength of the connection with other areas was weak.

## 4 Discussion

### 4.1 Research advantages

Reasonable transport planning should comprehensively consider the relationship between transportation, urban development, and environmental protection, which plays an important role in guiding the orderly and high-quality development of land space [42]. In the past, supporting urban development and meeting the need of residents have been goals of transport planning, which have increased transportation infrastructure to better serve the rapidly expanding cities [43]. Although previous studies have evaluated the impact of transportation construction on the ecological environment, little attention has been paid to ecological constraints in the preliminary transport planning [44]. In transport planning, the concept of combining ecology and development has been not well reflected, meanwhile, not well connected with other planning. In this study, a logical framework for the land suitability assessment was proposed to scientifically identify the layout and scale of traffic land for supporting transport planning.

Compared with previous studies, the first advantage is that we incorporated the bottom-line thinking to comprehensively evaluate the supporting capacity of resources and environment for traffic construction [45], which reflected the constraints and support of the environment on traffic construction from the aspects of land use, topography, ecological protection, and natural disasters. The second advantage is that we used the gravity model to analyze the strength of the connection between the city’s internal and external central nodes. Previous studies often regard the study area as a closed system, with less consideration of spillover effects. However, urban economic development is affected by the surrounding area [46]. We analyzed the connection strength between the internal central nodes, and the connection strength between the internal central nodes and the central nodes of the outer bordering area to comprehensively evaluate the influence of the socio-economic environment inside and outside the study area on urban development, which can more fully reflect the demand and necessity of traffic construction. The third advantage, from the perspective of ecological protection and food security, is to use restricted areas to modify the suitability of traffic construction. We paid more attention to the principle of resources constraint and ecological priority to promote the construction of ecological civilization and realize the harmony between humans and nature. The fourth advantage is that double evaluation is the scientific basis of national spatial planning, similar to the logical framework of this study. Therefore, national spatial planning and transport planning can be better connected and adapted.

### 4.2 Significance and new application

This study may be an innovative attempt at traditional transport planning evaluation under the background of the construction of ecological civilization and the new system of resource and environmental management, which embodies the change of the evaluation mode from post to synchronization. The distribution characteristics of land suitability for traffic planning are generally consistent with the existing highway distribution characteristics, which indicates that our research results are consistent with the reality to a certain extent. In the past, transportation planning and urban planning took economic development as the priority target, while less consideration was given to regional resource and environmental constraints, resulting in the disorderly development of the region [47]. Through resource and environment carrying capacity assessment and demarcation of restricted development zones, our study included not only thoughts of development, but also restrained urban disorderly expansion, which alleviated the conflict between development and protection in urban planning and traffic planning to some extent. Furthermore, the research results can provide important suggestions and measures for future transport planning. For example, based on the above results, we had the following suggestions. Although the highways in the Chengdu plain area were highly suitable, they caused serious damage to the environment. Therefore, the level of green construction and protection should be further improved. The area in the southeast of Sichuan is relatively close to the Yangtze River, Jinsha River, and other rivers, thus, the rivers should be protected during highway construction. The western part of Sichuan had many restrictions such as harsh terrain conditions and abundant ecological resources, which resulted in low suitability for construction in the west. However, there was still a highway with good suitability that was connected with Tibet. It is possible that the western development strategy and economic development needs were necessary for construction. Therefore, some harmless measures can be adopted to avoid damage to the ecological environment, such as increasing the proportion of bridges and tunnels. In northern Sichuan, the level of intensive use of expressways should be improved.

Methods proposed in this study such as the three-dimensional magic cube method can be used in the evaluation of resources and environment carrying capacity and the suitability of land space development, and can also provide a reference for the work of decision-makers or planners in the field of transport planning. They can also be used in other areas, of course, the following issues need to be paid attention to different targets.

Firstly, it is necessary to clarify whether the future development goal of the city focuses on ecological protection, economic development, or both are equally important. Based on different goals of urban development, the coupling standards of the demand for traffic construction and resource and environment carrying capacity, as well as the corrected coefficient of development restricted areas should be adjusted. Secondly, this study aimed to the evaluation of the suitability of expressway construction. Only an area with relatively good traffic conditions will be considered for highway construction. That is to say, the area with better traffic conditions will have stronger requirements for the construction of high-grade roads such as expressway. However, if demands for construction of other low-grade roads such as national or provincial roads are assessed, the better traffic conditions the lower the demand, so adjustments should be made according to goals of different levels of road construction. In addition, our study is only an initial template for suitability evaluation on highway construction, so it is not necessary to apply all the methods and index systems proposed in this study. According to local contexts, socio-economic conditions, and regional characteristics, related parameters such as key elements and the index system should be selected and adjusted. For example, the index system or parameters of resources and environment carrying capacity have differences between land or sea areas, between poor areas and developed areas [48, 49].

### 4.3 Limitations and prospect

Despite the advantages of this study, it also has some disadvantages. First of all, due to the limited data accuracy and the large area, based on the consideration of spatial heterogeneity, 500m was finally selected as the evaluation unit after multiple adjustments. However, the total width of most expressways is less than 100 meters [50]. The evaluation unit of this study is not tight enough to accurately select expressway units. Secondly, the evaluation unit setting and weights were subjective resulting in error propagation, but it can still provide a certain reference for expressway construction from a macro perspective. Furthermore, accuracy assessment has to be conducted to assess the classification results with available spatial data. Due to the lack of available classification data, accuracy assessment is difficult to carry out. However, the result of the suitability of expressway development was consistent with reality in Sichuan, which indicated that our method was reasonable to some extent. Therefore, we can collect more high-precision data and reduce the scale of the evaluation unit to improve research accuracy and applicability. Meanwhile, accuracy assessment is the research direction I need to improve in the future. Thirdly, local contexts of the region are dynamic, such as land use that changes all the time [51]. However, the double evaluation focuses more on static research, so it is difficult to accurately assess the supporting ability of resources and environment to transportation infrastructure in the future. Therefore, the next step of our study can try to select key indicators for prediction to dynamically assess the future land suitability. In addition, using existing results to make corrections, we selected the ecological protection red line and basic farmland as restricted areas to guarantee food and ecological security to a certain extent. However, the scope of the restricted area is not comprehensive enough, resulting in a weak security guarantee. In the future, we can construct an ecological security pattern [52], and select key elements and ecological protection red line as ecologically restricted areas. Furthermore, it is also possible to establish an index system of healthy farmland to comprehensively evaluate the quality of farmland [53], and select healthy farmland and basic farmland as agricultural restricted areas. To comprehensively select restricted areas is conducive to achieving sustainable urban development by expanding restricted areas. Finally, it still needs to further improve its applicability, for example, we can learn from the method of ecological corridor extraction that is the minimum cumulative resistance model [54]. Taking urban central nodes as the source and using results of development suitability as the resistance surface, a more precise transportation network will be extracted.

## 5 Conclusions

In this study, the concept and method of double evaluation of national spatial planning were introduced to the field of transport planning. To balance the relationship between urban development and ecological protection, we constructed a logical framework of resources and environment supporting, demands for traffic construction driving, and ecological protection red line and basic farmland constraining. The final land suitability was obtained based on the three-dimensional magic cube method. Our results showed that the overall carrying capacity was relatively high, showing the spatial distribution characteristics which were high in the east, north, and south, and low in the middle and west sides. The overall suitability of traffic construction presented a distributed pattern of high in the east and low in the west. The land suitability was relatively high, and the suitable areas with a grade from 8 to 10, accounted for 20.77% of the total study area, which could almost meet the demand for traffic land. The suitability of expressway development performed a circle structure with Chengdu City as the core and gradually decreasing to the periphery. The main findings in this study can effectively provide comprehensive and targeted support for decision-making in transport planning.
